# Expanding the positivity offset theory of anhedonia to the psychosis continuum

**DOI:** 10.1038/s41537-022-00251-x

**Published:** 2022-05-03

**Authors:** Marcel Riehle, Matthias Pillny, Tania M. Lincoln

**Affiliations:** Clinical Psychology and Psychotherapy, Institute for Psychology, Universität Hamburg, Hamburg, Germany

**Keywords:** Psychosis, Schizophrenia

## Abstract

People with schizophrenia and negative symptoms show diminished net positive emotion in low-arousing contexts (diminished positivity offset) and co-activate positive and negative emotion more frequently (increased ambivalence). Here, we investigated whether diminished positivity offset and increased ambivalence covary with negative symptoms along the continuum of psychotic symptoms. We conducted an online-study in an ad-hoc community sample (*N* *=* 261). Participants self-reported on psychotic symptoms (negative symptoms, depression, positive symptoms, anhedonia) and rated positivity, negativity, and arousal elicited by pleasant, unpleasant, and neutral stimuli. The data were analyzed with multilevel linear models. Increasing levels of all assessed symptom areas showed significant associations with diminished positivity offset. Increased ambivalence was related only to positive symptoms. Our results show that the diminished positivity offset is associated with psychotic symptoms in a community sample, including, but not limited to, negative symptoms. Ecological validity and symptom specificity require further investigation.

## Introduction

According to meta-analytic evidence, in-the-moment hedonic responding is intact in schizophrenia^1^. Nevertheless, people with schizophrenia, particularly those with motivational negative symptoms, show a marked reduction in goal-directed behavior^2–4^. This seems paradox^5^ given the pertaining view in emotion research that a primary function of emotional responding is to motivate adaptive behavior^6,7^. In a recent study, Strauss and colleagues^8^ therefore used advanced modelling of emotional responses in accordance with the Evaluative Space Model (ESM^9–11^) and detected alterations of in-the-moment hedonic responding in schizophrenia that had not been regarded in previous research. The ESM offers a framework for the mechanics underlying the formation of emotional experience that encompasses both discrete (e.g.,^12^), and dimensional (e.g.,^7,13^) accounts of emotion theory.

A core assumption of the ESM is that there are two partially separable emotional response systems that underlie *positivity* (i.e., hedonic, appetitive responding) and *negativity* (i.e., aversive, defensive responding). These two systems may co-activate, which explains the experience of *ambivalence*. Accordingly, ambivalence is the result of approach-avoidance conflicts which often result in behavioral inaction^10,14^. In this regard, Strauss and colleagues^8^ found that participants with schizophrenia and negative symptoms responded with *increased ambivalence* toward emotionally evocative stimuli, providing additional explanation for the passivity that is typical of negative symptoms.

Strauss and colleagues^8^ also found differences between people with and without schizophrenia for another marker of hedonic responding derived from the ESM, namely the *positivity offset*. The positivity offset concept is based on the ESM’s postulate of *activation functions* that underlie positivity and negativity. According to the ESM, positivity and negativity form as the result of evaluative processes that attempt to match (the experience of) autonomic arousal with emotional meaning^10^. However, the degree to which positivity and negativity activate as a function of arousal differs in that the negativity system deactivates almost entirely in low-arousing contexts, whereas the positivity system retains a certain level of activity. This surplus of positive over negative emotional activation in low-arousing contexts has been termed the positivity offset and is thought to motivate for exploratory behavior and curiosity^9^. The negativity system, on the other hand, shows a stronger response to increasing levels of arousal than the positivity system (steeper slope of the activation function). This has been termed *negativity bias*. In their study, Strauss and colleagues^8^ found that a diminished positivity offset predicted the severity of negative symptoms and self-reported anhedonia in schizophrenia.

The theoretic implication of these findings is that rather than a diminished hedonic *capacity* (the traditional definition of anhedonia), people with schizophrenia deactivate positive emotion too much in more neutral contexts which impedes the initiation of reward-seeking activity. However, if presented with sufficiently arousing pleasant stimuli, people with schizophrenia are able to activate normal levels of positivity, even though higher levels of concurrent negativity produce increased ambivalence and thereby diminish the pleasure experience^8^. Accordingly, the behavioral aberrations that are picked up as the negative symptoms of schizophrenia in contemporary interview-based assessments (e.g.,^15,16^) could be at least partially attributed to diminished positivity offset and increased ambivalence.

Whether *diminished positivity offset* and *increased ambivalence* can also inform early detection of vulnerability for negative symptoms or even approaches to prevention depends on whether or not these aberrations are distributed along the continuum of psychotic symptoms^17^. Because negative symptoms, specifically anhedonia, are a known risk factor for the transition from high-risk for psychosis to actual psychosis^18–20^ and because no effective treatments for negative symptoms in at-risk mental states have been identified^21^, a thorough understanding of the underlying mechanisms and their trajectories along the continuum is important.

Aberrations of in-the-moment emotional experience are currently not considered a continuous phenomenon that could underlie negative symptoms^22^. This is primarily because in attenuated negative symptoms (e.g., negative schizotypy), but not in schizophrenia, impaired hedonic responding to pleasant stimuli has been a reliable finding (i.e., the “schizophrenia spectrum anhedonia paradox”^23^). However, positivity offset and ambivalence are not contingent upon intact or impaired hedonic responding to pleasant stimuli^8^. Both could therefore represent consistent etiological factors for negative symptoms along the psychosis continuum.

In this study, we tested the hypothesis that a *diminished positivity offset* and *increased ambivalence* are associated with negative symptoms along the psychosis continuum. We expected that these associations would occur in addition to diminished positivity toward pleasant stimuli. To test for symptom specificity, we additionally accounted for potential associations with positive symptoms and depression.

## Results

### Sample characteristics

The sample characteristics are detailed in Table 1. Correlations of age, gender, and years of education, and symptom measures are shown in Supplementary Table S1.

**Table 1:** Sample characteristics (*N* = 261).

Measure	*M*/%	SD	Range
Age	41.3	13.7	19–75
% Male^a^	63.2		
Years of education	14.2	5.00	0–32
Employment status %			
Working/student full time	62.1		
Working/student part time	14.2		
marginally employed	6.9		
Unemployed	8.8		
Retired/on pension	8.1		
Marital/relationship status %			
Married (living with spouse)	40.6		
Married (living separated)	3.1		
Divorced	11.5		
Unmarried (in relationship)	18.4		
Unmarried (single)	25.3		
Widowed	1.2		
% picture set 1	47.9		
CAPE negative symptoms	2.00	0.57	1.00–3.62
CAPE depression symptoms	1.94	0.57	1.00–3.50
CAPE positive symptoms	1.69	0.61	1.00–3.65
CAS anhedonia	15.0	8.13	0–37

*CAPE* Community Assessment of Psychic Experiences^24^, *CAS* Chapman physical and social anhedonia scales (sum score)^25,26^.

^a^No participant answered “other/unidentified”; 1 participant did not answer the question.

### Raw emotional responses

**Fig. 1:**
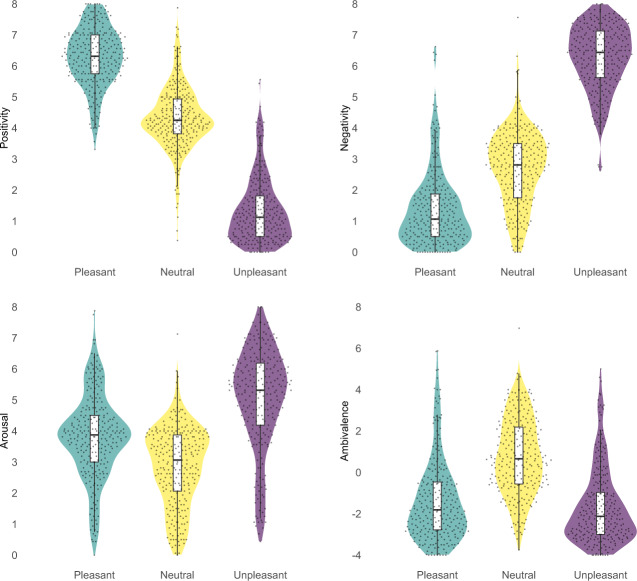
Violin plots for raw emotional responses (positivity, negativity, arousal) and ambivalence with box-plots and superimposed individual participant data points. The box-plots illustrate the first and third quartile (box limits), median (center line), and ±1.5 interquartile range (whiskers). Individual data points correspond to the mean response of each participant for the respective stimulus category and response scale.

In a first step, we tested for differences in raw emotional responses (i.e., positivity, negativity, arousal) between pleasant, neutral, and unpleasant pictures without the inclusion of symptom scales as moderator. Figure 1 illustrates the distribution of values over the three stimulus categories for each response variable as estimated via the respective multilevel linear models (MLM). The full models are reported in the Supplementary Tables (positivity: Table S4, negativity: Table S8, arousal: Table S14). For all three response variables, there were significant main effects of stimulus category, all *F*s ≥ 49.97, *p*s < 0.001. Within each response variable, all stimulus category differences were significant with *β*s ≥ 0.39, *t*s ≥ 4.91, *p*s < 0.001. These differences were all in the expected direction (e.g., more positivity for pleasant pictures), except that unpleasant pictures produced more arousal than pleasant pictures, *b* = 1.22, *β* = 0.51, 95% CI [0.29, 0.73], *t*_*(198.4)*_ = 5.61, *p* < 0.001.

**Fig. 2:**
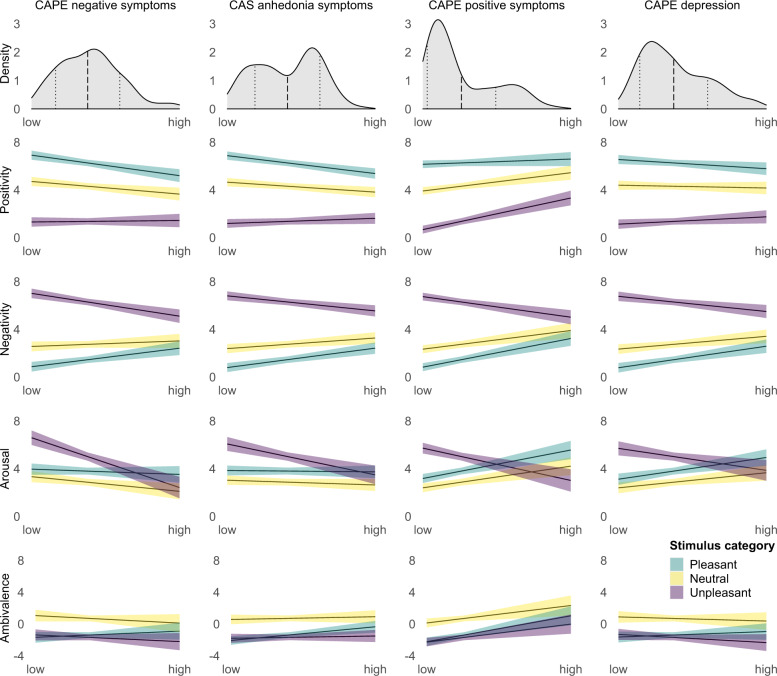
Sample distribution of symptom scale scores and moderator effects of each symptom scale on positivity, negativity, arousal, and ambivalence for each picture category based on the MLM. The top row shows the sample distribution of the scores for each symptom scale (density function estimated with 1/30 observations, thus 8–9 participants per data point). The dashed lines represent the mean, the dotted lines ±1 SD. In the 4 (response scale) by 4 (symptom measure) line plots, each line corresponds to the effect of a given moderator within a given stimulus category. The shaded error bars illustrate the 95% CI. The “low” and “high” endpoints of the *x*-axis correspond to the observed minimum and maximum values for the respective symptom measure (cf. Table 1).

We then tested for moderation of these category differences by each symptom scale. As described in detail in the methods section, separate MLMs were calculated for each symptom scale (i.e., for each moderator) and each response scale. Each of these models included the target moderator (e.g., CAPE negative symptoms, CAS anhedonia) and the other two symptom areas (in the example CAPE positive symptoms and CAPE depression) as covariates. For each of the three raw response variables (positivity, negativity, arousal) we found significant stimulus category by symptom measure interactions for each of the four symptom measures (CAPE negative symptoms, CAPE depression, CAPE positive symptoms, and CAS anhedonia), all *F*s ≥ 7.19, *p*s ≤ 0.001. The effects of the moderators on raw emotional responses within each stimulus category are illustrated in Fig. 2 and reported with test statistics in Supplementary Table S2. Full model results and effects without covariates are also reported in the Supplementary material. Figure 2 contains the following significant effects:

#### Positivity

Higher scores on CAPE negative symptoms and CAS anhedonia predicted *decreased* positivity in response to pleasant and neutral stimuli. Higher scores on CAPE positive symptoms predicted *increased* positivity in response to neutral and unpleasant stimuli.

#### Negativity

Higher scores on all four symptom measures predicted *decreased* negativity in response to unpleasant and *increased* negativity in response to pleasant stimuli. All symptom measures except CAPE negative symptoms also predicted *increased* negativity in response to neutral stimuli.

#### Arousal

Higher scores on all four symptom measures predicted *decreased* arousal in response to unpleasant stimuli. CAPE negative symptoms predicted *decreased* arousal also for neutral stimuli, whereas CAPE positive symptoms and depression predicted *increased* arousal in response to neutral and pleasant stimuli.

### Ambivalence

Again, in a first step, we tested for differences in ambivalence between stimulus categories without the inclusion of moderators. The results of this MLM are also illustrated in Fig. 1 and showed that ambivalence was higher for neutral than for unpleasant, *b* = 2.39, *β* = 0.77, 95% CI [0.60, 0.94], *t*_*(135.9)*_ = 10.94, *p* < 0.001, and pleasant stimuli, *b* = 2.08, *β* = 0.67, 95% CI [0.50, 0.84], *t*_*(130.9)*_ = 9.64, *p* < 0.001, and that there was no difference between pleasant and unpleasant stimuli, *b* = 1.22, *β* = −0.10, 95% CI [−0.26, 0.06], *t*_*(116.4)*_ = 1.48, *p* = 0.143.

We then used the same approach to analyze the moderating effect of symptoms on these category differences as described for raw emotional responses. We found significant stimulus category by symptom measure interactions for each of the four symptom measures, all *p*s ≤ 0.006. The only significant effects within stimulus categories shown for ambivalence in Fig. 2 (and reported in Supplementary Table S2) were that CAS anhedonia predicted increased ambivalence for pleasant stimuli, *b* = 2.00, *β* = 0.12, 95% CI [0.05, 0.20], *t*_*(311.6)*_ = 3.15, *p* = 0.002, and that CAPE positive symptoms predicted increased ambivalence in all three stimulus categories (pleasant: *b* = 3.71, *β* = 0.24, 95% CI [0.13, 0.36], *t*_*(282.7)*_ = 4.19, *p* < 0.001; neutral: *b* = 2.50, *β* = 0.16, 95% CI [0.04, 0.28], *t*_*(305.0)*_ = 2.67, *p* = 0.008; unpleasant: *b* = 2.57, *β* = 0.17, 95% CI [0.05, 0.28], *t*_*(277.1)*_ = 2.86, *p* = 0.005). The main effect of CAPE positive symptoms was significant, *F*_*(1248.8)*_ = 11.42, *p* < 0.001. The full MLM results are reported in Supplementary Tables S19–23.

### Positivity offset and negativity bias

As described in more detail in the methods section, the positivity offset was estimated as the difference in intercepts of the positivity and negativity activation functions (i.e., positivity/negativity when arousal = 0). Thus, in the respective MLM, we combined positivity responses to pleasant and neutral stimuli and negativity responses to unpleasant and neutral stimuli to a single dependent variable *emotional activation*. The model then included the predictors activation function (positivity vs. negativity), arousal (coded as 0–8), and their interaction. Thus, the main effect of activation function corresponded to the positivity offset. The difference between the two activation functions in slopes of arousal predicting emotional activation served as the test for the negativity bias. Variances for positivity offset and negativity bias were estimated via random slopes.

As for the other analyses, we first tested a model without moderators. In this base positivity offset MLM, we found a significant positivity offset and a significant negativity bias. The intercepts for the positivity and negativity activation functions were estimated at 5.29, 95% CI [4.94, 5.64] and 2.89, 95% CI [2.54, 3.24], respectively. The difference between these two intercepts, the positivity offset, was a random effect in the model with a mean of 2.40, SD = 0.92, and was significant, *β* = 0.98, 95% CI [0.78, 1.18], *t*_*(183.2)*_ = 9.64, *p* < 0.001. The slope of the negative, but not the positive, activation function was significant (conditional effects of arousal within activation function). For positivity it was estimated at *b* = 0.01, *β* = 0.01, 95% CI [−0.02, 0.04], *t*_*(644.3)*_ = 0.75, *p* = 0.456, and for negativity at b = 0.42, *β* = 0.41, 95% CI [0.38, 0.44], *t*_*(584.0)*_ = 26.00, *p* < 0.001. The difference between these slopes, the negativity bias, was a random effect in the model with a mean of 0.41, SD = 0.17 and was significant, *β* = 0.40, 95% CI [0.36, 0.45], *t*_*(616.5)*_ = 17.56, *p* < 0.001.

**Fig. 3:**
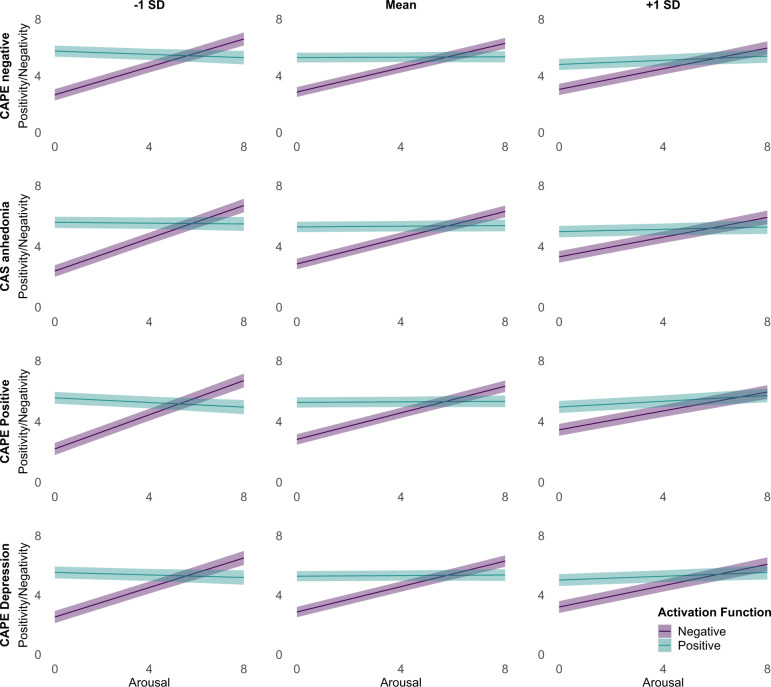
Positivity and negativity activation functions estimated via MLM for different levels of the symptom measures. The shaded error bars represent the *95% CI*.

When including symptom measures as moderators in the next step, there were significant activation function by symptom measure interactions and significant three-way interactions of activation function by arousal by symptom measures for all four symptom measures, all *p*s < 0.001. As can be seen in Fig. 3 and Table 2, these results signified a diminished positivity offset and diminished negativity bias at higher symptom levels. The full MLM results are reported in Supplementary Tables S24–S28.

**Table 2:** Estimates of positivity offset and negativity bias for different levels of symptoms and overall effect of symptoms on positivity offset and negativity bias.

Scale	Positivity offset	*β* (*95% CI*)	Negativity bias	*β* (*95% CI*)
CAPE negative				
−1 SD	3.09	−0.27 (−0.35, −0.19)	0.55	−0.13 (−0.17, −0.08)
Mean	2.43		0.41	
+1 SD	1.77		0.29	
CAS anhedonia				
−1 SD	3.21	−0.32 (−0.40, −0.23)	0.55	−0.13 (−0.17, −0.09)
Mean	2.44		0.41	
+1 SD	1.67		0.28	
CAPE positive				
−1 SD	3.37	−0.38 (−0.46, −0.30)	0.64	−0.21 (−0.25, −0.17)
Mean	2.44		0.41	
+1 SD	1.50		0.22	
CAPE depression				
−1 SD	3.01	−0.24 (−0.33, −0.24)	0.55	−0.12 (−0.16, −0.08)
Mean	2.42		0.41	
+1 SD	1.82		0.29	

*N* = 261. In all models covariates were age, gender, years of education, and other symptom domains. Positivity offset refers to the difference in intercepts of the positive and negative activation function for arousal = 0. Negativity bias refers to the difference in slopes between the negative and positive activation function. All values for positivity offset and negativity bias are significant differences (all *p*s < 0.001). Beta-values correspond to the standardized effect of the respective symptom scale on the positivity offset and negativity bias, respectively, in the model underlying the estimation of the different values.

*CAPE* Community Assessment of Psychic Experiences, *CAS* Chapman Anhedonia Scales.

## Discussion

In this online-study, we investigated in a community sample (*N* = 261) whether the positivity offset theory of anhedonia in schizophrenia^8^ can be expanded to negative symptoms in the psychosis continuum.

Our first hypothesis, that there would be an association between higher levels of negative symptoms and a diminished positivity offset, was supported by our data. Together with the findings of Strauss et al.^8^, this suggests that diminished activation of positivity in low-arousing contexts is a mechanism that consistently underlies negative symptoms along the psychosis continuum. However, positive symptoms and depression were also associated with the diminished positivity offset. This suggests an association with psychosis proneness or psychopathology in general which needs further study (see below). The primary function of a normative positivity offset is to motivate for exploratory behavior. Therefore, an interesting avenue for future research is to investigate whether the diminished positivity offset adds to explaining diminished goal-directed activity that has been found in ecological momentary assessment (EMA) studies both in people with schizophrenia^2–4,27^ and in people with elevated levels of psychosis proneness^28,29^.

An unexpected finding in our positivity offset analyses was that increasing levels of arousal predicted increasing positivity only at higher levels of psychotic symptoms with no significant relationship at low or moderate levels (cf. Fig. 2). Strauss et al^8^. found positive associations both for people with and without schizophrenia with a stronger (more positive) association for people with schizophrenia. Thus, our findings appear to replicate the moderating effect of higher levels of psychotic symptoms on the association of arousal and positivity, while at the same time suggesting a scaled-back basis for the association of arousal and positivity. One likely reason for this is that our participants rated the pleasant pictures as less arousing than the participants in the IAPS validation study^30^ (our sample: *M* = 3.80, SD = 2.40; validation data: *M* = 5.07, SD = 2.34, Cohen’s *d* = −0.54) while rating the neutral pictures in a manner comparable to the validation participants for the neutral pictures (our sample: *M* = 2.87, SD = 1.93; validation data: *M* = 3.03, SD = 1.93, *d* = −0.08). Diminished arousal ratings for pleasant IAPS pictures have been observed in other studies comparing samples from Germany (such as ours) to US samples (such as the sample of Strauss et al.^8^)^31–33^, which could indicate a cross-cultural difference.

We had also hypothesized an association between higher levels of negative symptoms and increased ambivalence. This hypothesis was not supported by our data: Positive but not negative symptoms were associated with increased ambivalence for pleasant, neutral, and unpleasant stimuli. In Strauss and colleagues’ study^8^, ambivalence was associated with negative symptoms in schizophrenia but not in controls. Together, this could imply that ambivalence is associated with negative symptoms only in schizophrenia. This could be the case, for example, if the association was detectable only at higher symptom levels found primarily in people with schizophrenia or if primary or secondary factors associated with the disorder (e.g., specific pathophysiology, treatment effects, etc.) accounted for the discrepant findings. However, Strauss and colleagues^8^ did not report on positive symptoms. It therefore remains unclear whether their association of negative symptoms and increased ambivalence in schizophrenia would have remained significant if they had controlled for positive symptoms. In our study, negative symptoms were also associated with increased ambivalence once we removed the covariates (including positive symptoms) from the model (see Supplementary Table S3). Another interpretation therefore is that increased ambivalence is not related to negative but to positive symptoms. It is worth noting that increased ambivalence in schizophrenia is likely contingent upon elevated activation of “inadequate” emotion (e.g., more positivity in unpleasant contexts) with activation of “adequate” emotion remaining largely intact^1,8,34,35^. In our analyses of the raw emotional responses, only positive symptoms were associated with both increased positivity for unpleasant and increased negativity for pleasant stimuli which further underscores the association of positive symptoms and increased ambivalence.

Attenuated negative symptoms were associated with diminished raw emotional responses for both pleasant and unpleasant stimuli when controlling for positive symptoms and depression. This replicates several previous findings in psychosis-prone and at-risk mental state samples^36–42^, including studies that controlled for depression^43–45^. Nevertheless, these findings for attenuated negative symptoms concord with findings of diminished emotional reactivity to pleasant and unpleasant stimuli in major depression^46–49^.

In major depression, the diminished emotional reactivity for pleasant and unpleasant stimuli is interpreted as a correlate of a generalized amotivational state that emerges in response to adverse situations in which continued activity would be detrimental^46–49^. In such situations, not experiencing strong emotions that motivate for action would be adaptive^50^. Similar hypotheses have been proposed for negative symptoms, highlighting the putatively causal roles of repeated or extreme personal failure^51^ and social defeat^52^ (e.g., asociality as an adaptive coping mechanism in response to constant social rejection^52,53^). The inactivity goal might be achieved implicitly by the organism by reducing the sensitivity to changes in emotional context^54^. This hypothesis has been termed *emotion* *context* *insensitivity*^46^. Diminished emotional reactivity in emotional experience tasks, such as the one used here, has been considered to back up the idea of emotion context insensitivity^47–49^. Interestingly, among the symptoms of major depression, anhedonia has been found to produce this pattern of results rather than depressed mood^49^, which may explain why we found these associations for attenuated negative symptoms when controlling for depression, but not vice-versa. This overlap with major depression is indicative of the transdiagnostic nature of negative symptoms in general and anhedonia in particular^22,55^. Findings showing that people with schizophrenia^56,57^ and people in at-risk mental states^58,59^ appear to have difficulty in differentiating between positive and negative emotional states further support the assumption that emotion context insensitivity plays a role at various severity levels of the psychosis continuum.

Such blurring of the boundaries between positive and negative emotional experience could not only explain diminished overall emotional activation, but also increased ambivalence and a diminished positivity offset. On the one hand, our data suggest that more differentiation is necessary, because diminished emotional activation was specifically associated with negative symptoms, increased ambivalence specifically with positive symptoms, and the diminished positivity offset unspecifically with all assessed symptom areas. On the other hand, our data also suggest that all interpretations of symptom-specificity should be made with caution. There were high correlations between the various symptom scales (particularly between the CAPE scores, *r*s = 0.72–0.79; Table S1) which may have led to suppressor effects and subsequently to underestimation of the associations. The overlap of symptom domains is a likely artifact of our reliance on a self-report measure of the lifetime prevalence of psychotic-like experiences. Future research therefore should further investigate symptom-specificity and use interview-based assessments of current symptom levels. In this regard, future work should also investigate to which degree processes such as psychophysiological responding, interoceptive awareness (e.g., alexithymia), or expectations, such as low-pleasure or demotivating beliefs^60–62^, play a role in explaining diminished emotional experience, increased ambivalence, and diminished positivity offset.

Another important question is to which degree our results generalize across the psychosis continuum. This question is strongly related to the question of symptom severity and variation in our sample. Our community sample’s CAPE and CAS scores (see Table 1 and Fig. 1) were within the range of samples at-risk for psychosis^63–67^ and other community samples^68–72^, and higher than in all-healthy control samples^26,40,65,73–77^. The scores also covered a broad range for each symptom domain. The data suggest considerable variation from very low symptoms to very high and likely clinically relevant symptoms^69,78^. Therefore, we are confident that our sample covered a broad range of the psychosis continuum and that our findings are likely to generalize to samples at its more severe end.

Several strengths and limitations of our study need to be considered when interpreting our results. A strength is that we estimated all parameters using MLMs which allowed us to model stimulus category (level 1) by symptom severity (level 2) interactions that cannot be modelled in repeated measures ANOVA on averaged responses across stimuli^79^. Accordingly, Strauss et al.^8^ did not report associations of negative symptoms with raw emotional responses within stimulus categories. Another advantage of our analytic approach is that we analyzed positivity offset and ambivalence on the level of single stimuli as opposed to the approach taken by Strauss et al.^8^, who analyzed these concepts on the basis of stimulus category means. Ours is also the first study we are aware of that modelled positivity offset and negativity bias via MLM. Another strength is that we were able to analyze a relatively large sample which provided sufficient power to test our hypotheses (small effect sizes, *f²* = 0.02, of single predictors in our models could be detected with a power of 92% as indicated by simulation-based power analysis^80^). A limitation is that we had to rely on self-report for all measures due to collecting the data online. Interview-based symptom assessments and inclusion of other levels of the emotional response (psychophysiology, behavior) would be worthwhile additions to our research design. Another limitation related to the online data collection is that we do not know to which degree participants followed our instructions correctly. Despite excluding 3% of the sample with long-string answers, the signal-to-noise ratio in our data may have been low. However, the high degree of overlap of our findings for raw emotional responses with those of previous research in the psychosis continuum mentioned above indicate sufficient data quality. Finally, even though our sample certainly fulfilled several diversity criteria due to posing little restrictions on participation, we did not collect a representative sample, which limits the generalizability of our results to the general population.

In conclusion, our data support a dimensional model for the positivity offset theory of anhedonia^8^ even though the lack of symptom specificity for negative symptoms requires further study. Overall, our findings point toward a more general difficulty in the differentiation between positive and negative emotional states underlying the in-the-moment emotional aberrations found across the psychosis continuum. In order to establish diminished positivity offset and increased ambivalence as potential targets of early detection and intervention, our findings need replication in youth and young adults with at-risk mental states and in people with first-episode psychosis.

## Methods

### Recruiting procedure

We conducted an online-study in an ad-hoc convenience sample. Participants were either micro-workers recruited via figure-eight.com (formerly crowdflower.com), as in earlier work by our group^68,81,82^, or participants recruited within the extended private networks of the researchers involved. We instructed participants to complete the study on a laptop, PC, or tablet at a time when they were undisturbed. Micro-workers were reimbursed with 0.95 US $ for their participation. Other participants were offered to take part in a lottery for a coupon for an online retailer. All participants provided informed written consent before partaking in the study. For this, participants received a standardized on-screen study information and consent form detailing all aspects of the study, rights and consequences of participation, and contact information of the principal investigator. Participants were instructed to download this form as a PDF and then had to check a check box indicating they had read all information and consented to participate before being able to proceed with the study. The study’s procedures were approved by the local ethics committee of the University of Hamburg, Hamburg, Germany (AZ: 2017_129 Riehle) and conducted in accordance with the latest version of the Declaration of Helsinki.

An inclusion criterion for all participants was an age of at least 18 years and they had to indicate sufficient German language skills. Micro-workers additionally had to be “level 2 contributors” (i.e., “more experienced, higher accuracy contributors”), living in Austria, Germany, Luxembourg, Liechtenstein, or Switzerland.

The study was implemented in EFS Survey software (Questback GmbH, 2017). The survey was accessed by 521 people of whom 393 provided informed consent to participate. Of these, 269 participants provided eligible data-sets (i.e., completing questionnaire assessments and at least 75% of the emotional experience task). There were no missing data due to a forced entry format and all participants providing eligible data sets completed 100% of the emotional experience task. Eight of these data sets (3%) had to be removed due to long-strings (i.e., more than 50 similar answers in a row^83^). This left us with *N* = 261 (77% recruited via crowdflower) individual data-sets available for analysis.

### Materials and instruments

#### Symptom measures

We assessed life-time frequency of negative symptoms, depression, and positive symptoms with the three respective scales of the Community Assessment of Psychic Experiences (CAPE^24^; cape42.homestead.com). The CAPE comprises 42 items (e.g., “Do you ever feel that you are lacking in motivation to do things?” [negative symptoms]) that are answered on a Likert-scale ranging from 1 (“never) to 4 (“nearly always”). We calculated mean values for each scale with higher scores indicating more frequent psychotic symptoms.

As an additional measure of attenuated negative symptoms, and in keeping with Strauss and colleagues^8^, we assessed anhedonia with the 43-item German version of the revised Chapman Physical and Social Anhedonia Scales (CAS^25,26^). The CAS comprises 43 items (e.g., “Dancing, or the idea of it, has always seemed dull to me”) that are answered on a dichotomous 0 = “disagree”/1 = “agree” scale. We calculated a single sum score for physical and social anhedonia, with higher scores indicating higher anhedonia.

#### Emotional experience task

The emotional experience task was based on the one used by Strauss and colleagues.^8^ We randomized participants to one of two parallel sets of 48 emotionally evocative pictorial stimuli (16 pleasant, 16 unpleasant, 16 neutral) taken from the International Affective Picture System^84^. Both sets had the following properties: Within each stimulus category, one-half of the stimuli depicted social scenes, the other half depicted nonsocial scenes. Selected stimuli differed in normative valence (pleasant > neutral > unpleasant) and arousal (pleasant = unpleasant > neutral). There were no overlaps or significant differences in normative valence or arousal between the two sets. The IAPS picture numbers in set 1 were: 1051, 1201, 1270, 1280, 1460, 1590, 1721, 2080, 2091, 2200, 2208, 2210, 2385, 2440, 2518, 2710, 2720, 2840, 3010, 4660, 5270, 5395, 5510, 5731, 5740, 5779, 5830, 5910, 6020, 6540, 6834, 7100, 7185, 7190, 7205, 7470, 7580, 8030, 8120, 8300, 9181, 9210, 9220, 9300, 9433, 9470, 9561, 9910. The IAPS picture numbers in set 2 were: 1230, 1302, 1710, 1722, 1750, 2057, 2190, 2391, 2495, 2500, 2514, 2516, 2800, 2880, 2890, 2900, 3015, 4250, 4611, 4652, 5120, 5130, 5260, 5410, 5480, 5535, 5600, 5875, 6210, 6350, 6800, 7004, 7150, 7175, 7217, 7270, 7481, 8185, 8190, 8480, 8490, 9041, 9373, 9410, 9570, 9600, 9630, 9911.

The emotional experience task was implemented after participants had answered the questionnaires. The 48 trials, one for each stimulus, were presented in a random sequence. In each trial, participants rated their positivity (“How positive does this picture make you feel?”), negativity (“How negative does this picture make you feel?”), and arousal (“How calm/excited does this picture make you feel?”), respectively. Each rating was made on a 9-point scale that ranged from 0 (“not at all” [”very calm” for arousal]) to 8 (“very much” [”very excited” for arousal]). Matching self-assessment manikins^85^ were presented at the scales’ poles. Each scale was presented on a separate slide and below the stimulus. Participants had to press a “proceed”-button after each rating, thereby calling the next rating (or the next trial).

#### Data analysis

As recommended for data with multiple responses nested within participants^79^, we used MLMs to test our hypotheses. For all dependent variables, we first estimated a base-model that tested for differences in emotional responses (i.e., between stimulus categories or between activation functions) and then estimated four separate models in a second step that each included one of the symptom measures (i.e., CAPE negative symptoms, CAPE depression, CAPE positive symptoms, or CAS anhedonia) as a level-2 moderator of these differences. To increase the comparability across symptom measures, we divided all questionnaire scores by their respective possible scale maximum for these analyses, so that all scores were expressed with a similar range (0–1). In addition, all scores were centered to the respective sample mean. All models included gender, age, and years of education as covariates. To control for overall psychosis proneness and general psychopathology, all models testing for moderation by a given symptom measure additionally included the respective other symptom domains as covariates (e.g., a model focusing on the moderation of CAPE negative symptoms included CAPE positive symptoms and CAPE depression as covariates). Analyses were conducted in IBM SPSS 26. We report effect sizes as standardized *β* and tested significance on a two-sided 5% α-level, corrected for multiple tests using Bonferroni correction.

#### Raw emotional response differences between stimulus categories

We first tested for differences in raw emotional responses (the raw values of positivity, negativity, and arousal for each stimulus) between stimulus categories. Separate models were calculated for each of these three dependent variables, in which we specified a fixed effect for stimulus category (pleasant vs. neutral vs. unpleasant). We modelled by-subject random intercepts, by-subject random slopes for stimulus category, and by-stimulus random intercepts within each stimulus category (i.e., maximal models^79^).

In the second analysis step, we additionally included fixed effects for the main effect of a given symptom measure and for its interaction with stimulus category. Conditional effects, i.e., the effect of a given moderator within each of the three stimulus categories, were calculated to follow-up interactions that showed at least trend-level significance (*p* < 0.10).

#### Ambivalence

We used the following formula to calculate ambivalence: $Ambivalence = \frac{{Positivity + Negativity}}{2} - |Positivity - Negativity|$ (cf.^86–88^). This formula combines the two core components of ambivalence (cf.^88^): The first term captures the strength of the ambivalent response (labelled as “total affect” by Kaplan^88^). High levels of co-activated emotion are thought to represent the distressing aspect of ambivalence^14^ as opposed to situations where both positivity and negativity are very low (i.e., indifference). The second term captures the degree of matching between positivity and negativity (labelled as “polarity” by Kaplan^88^). Therefore, it is a measure of mere co-activation that is needed to differentiate between strong, but polarized, emotional responses and strong, but non-polarized, emotional responses. The formula yields values ranging between −4 and 8 with higher values corresponding to higher degrees of ambivalence. For example, positivity and negativity ratings of 8 and 2, respectively, would yield an ambivalence value of −1, ratings of 5 and 5, respectively (same total affect as in first example), an ambivalence value of 5, and ratings of 6 and 0, respectively (same polarity as in first example), an ambivalence rating of −3. In contrast to previous research that calculated ambivalence scores based on individual mean ratings across all stimuli^8,86^, here we calculated ambivalence scores for each stimulus separately. We submitted these values as the dependent variable to an MLM analysis that used the same approach as for raw emotional responses (i.e., two analysis steps and same set of predictors/moderators).

#### Positivity offset and negativity bias

In line with previous studies^8,9^, we used regression parameters to model positivity offset and negativity bias. Accordingly, we regressed positivity and negativity on arousal. However, instead of linear regression^8^, we used MLM to estimate the positivity and negativity activation functions and thereby positivity offset and negativity bias. The dependent variable in this MLM was “emotional response” (i.e., positivity *or* negativity) which was predicted by a dichotomous factor positivity vs. negativity activation function, by arousal, and their interaction. For the positivity activation function, we used the positivity and arousal responses to the 16 pleasant and 8 neutral stimuli and for the negativity activation function, we used the negativity and arousal responses to the 16 unpleasant and 8 neutral stimuli. We split the neutral category randomly per participant so that one half each represented responses for the positivity and negativity activation function for low-arousing stimuli. This was done so that no stimulus was included twice in the analysis, as is a requirement for being able to meaningfully assess a by-stimulus random intercept. In addition to the fixed effects, we included by-subject random slopes for activation function and for the activation function by arousal interaction and a by-stimulus random intercept^79^. The fixed effect of activation function provides information on the mean difference between the intercepts of the two activation functions (i.e., when arousal = 0) across participants and therefore a statistical test of the positivity offset. The activation function by arousal interaction provides information on the mean difference in slopes of the two activation functions across participants and therefore a statistical test of the negativity bias. After calculating this base-model, we estimated separate models, each including one of the symptom measures as a level-2 moderator. Accordingly, we added a main effect for the symptom measure and all possible interaction effects. The interaction of interest was the activation function by symptom measure interaction because it signified whether the difference in activation function intercepts (i.e., the positivity offset) would be moderated by the symptom measure. We also explored the significance of the three-way interaction of activation function by arousal by symptom measure, since this tested for moderation of the difference in the two activation functions’ slopes (i.e., the negativity bias).

## Supplementary information

Additional Results Tables

## Data Availability

The data of this study are available for scientific use via psycharvies.org at10.23668/psycharchives.5673. The analysis code for the multilevel models calculated in the manuscript is available via psycharchives.org at10.23668/psycharchives.5674.
