# Supplementary material for: Expanding the positivity offset theory of anhedonia to the psychosis continuum
Source: Schizophrenia (Heidelb). 2022 May 3;8(1):47. doi: 10.1038/s41537-022-00251-x (PMC9261090; doi:10.1038/s41537-022-00251-x)
Supplement: Supplementary file 1 — Additional Results Tables [file 41537_2022_251_MOESM1_ESM.pdf]

Supplementary material for

„Expanding the positivity offset theory of anhedonia to the psychosis spectrum"

Marcel Riehle, Matthias Pillny, & Tania M. Lincoln

Clinical Psychology & Psychotherapy, Institute for Psychology, University of Hamburg, Hamburg, Germany

Correspondence should be addressed to:

Dr. Marcel Riehle, marcel.riehle@uni-hamburg.de

Contents of this supplement

Correlations of symptom measures and covariates (Table S1) 3

Analyses of raw emotional responses and ambivalence... 4

Results of moderator analyses for raw emotional responses and ambivalence (Table S2) 4

Results of moderator analyses for raw emotional responses and ambivalence without covariates (Table S3).. 6

Positivity: Full models (Tables S4-S8) 7

Negativity: Full models (Tables S9-S13) 12

Arousal: Full models (Tables S14-S18)... 17

Ambivalence: Full models (Tables S19-S23)... 22

Positivity offset analyses.. 27

Full positivity offset models (Tables S24-S28)... 27

Supplementary positivity offset figures (Figures S1-S3) 36

Correlations of symptom measures and covariates (Table S1)

Table S1. Correlations of psychosis proneness measures with age, sex, and years of education, and with each other.

	CAPE negative	CAPE depression	CAPE positive	CAS anhedonia
Age	-.46***	-.39***	-.56***	-.46***
Gender	-.16*	.07	.07	-.07
Years of education	-.28***	-.30***	-.39***	-.30***
CAPE negative		.79***	.72***	.56***
CAPE depression			.76***	.45***
CAPE positive				.46***

Note: *N* = 261. CAPE = Community Assessment of Psychic Experiences; CAS = Chapman Physical and Social Anhedonia Scales. Gender is coded with male=0, female=1.

* *p*<.05, *** *p*<.001

Analyses of raw emotional responses and ambivalence

Results of moderator analyses for raw emotional responses and ambivalence (Table S2)

Table S2. *Unstandardized effect estimates (based on MLM) of the symptom measures within each stimulus category for positivity, negativity, arousal, and ambivalence.*

**DV** Stimulus Category	CAPE negative symptoms [a]			CAS anhedonia [a]			CAPE positive symptoms [b]			CAPE depress. Symptoms [c]		
	b	*t*	*p*	*b*	*t*	*p*	*b*	*t*	*p*	*b*	*t*	*p*
**Positivity**												
Pleasant	**-1.97**	**-4.52**	**<.001**	**-1.74**	**-5.15**	**<.001**	0.50	1.21	.227	-0.93	-2.07	.039
Neutral	**-1.22**	**-2.80**	**.005**	**-0.96**	**-2.85**	**.005**	**1.74**	**4.23**	**<.001**	-0.28	-0.63	.529
Unpleasant	0.14	0.31	.758	0.50	1.36	.175	**3.01**	**7.02**	**<.001**	0.74	1.58	.116
**Negativity**												
Pleasant	**1.78**	**3.79**	**<.001**	**1.89**	**4.89**	**<.001**	**2.73**	**6.30**	**<.001**	**2.17**	**4.50**	**<.001**
Neutral	0.53	1.11	.268	**1.03**	**2.60**	**.010**	**1.78**	**3.99**	**<.001**	**1.29**	**2.62**	**.009**
Unpleasant	**-2.20**	**-4.78**	**<.001**	**-1.47**	**-3.88**	**<.001**	**-1.96**	**-4.76**	**<.001**	**-1.53**	**-2.08**	**.001**
**Arousal**												
Pleasant	-0.51	-0.86	.390	-0.13	-0.31	.759	**2.71**	**4.89**	**<.001**	**2.19**	**3.60**	**<.001**
Neutral	**-1.41**	**2.47**	**.014**	-0.46	-1.12	.264	**2.06**	**3.83**	**<.001**	**1.53**	**2.59**	**.010**
Unpleasant	**-4.80**	**-6.14**	**<.001**	**-3.01**	**-4.37**	**<.001**	**-3.08**	**-4.49**	**<.001**	**-2.22**	**-2.82**	**.005**
**Ambivalence**												
Pleasant	0.89	0.957	.340	**2.00**	**3.15**	**.002**	**3.71**	**4.19**	**<.001**	0.83	0.89	.391
Neutral	-1.08	-1.10	.274	0.40	0.55	.580	**2.49**	**2.67**	**.008**	-0.63	-0.61	.540
Unpleasant	-0.97	-1.02	.309	0.33	0.51	.614	**2.57**	**2.86**	**.005**	-1.24	-1.27	.206

Note: N = 261. DV = dependent variable; CAPE = Community Assessment of Psychic Experiences; CAS = Chapman Physical and Social Anhedonia Scales. Questionnaires were normalized (range 0-1). Scale range for Positivity, Negativity, Arousal = 0-8; for Ambivalence = -4-8. In all models covariates were age, gender, years of education, and other symptom domains (cf. superscripts). All effects with $p < .016$ were significant after Bonferroni correction for 3 tests (printed bold).

[a] add. covariates: CAPE positive, depression. [b] add. covariates: CAPE negative, depression. [c] add. covariates CAPE negative, positive.

Results of moderator analyses for raw emotional responses and ambivalence without covariates (Table S3)

Table S3 Unstandardized conditional effects (based on MLM) of the moderators within each stimulus category for positivity, negativity, arousal ratings, and ambivalence. <u>*No covariates*</u> were included in the models (full models not reported).

**Stimulus Category**	**CAPE negative symptoms**			**CAPE depress. symptoms**			**CAPE positive symptoms**			**CAS anhedonia**		
	b	***t***	***p***	***b***	***t***	***p***	***b***	***t***	***p***	***b***	***t***	***p***
						Positivity						
Pleasant	**-1.083**	**-3.455**	**.001**	-0.601	-1.910	.057	-0.632	-2.163	.031	**-1.405**	**-4.559**	**<.001**
Neutral	-0.371	-1.155	.249	-0.003	-0.009	.993	0.535	1.805	.072	-0.678	-2.120	.035
Unpleasant	**0.993**	**2.726**	**.007**	**1.026**	**2.843**	**.005**	**1.810**	**5.613**	**<.001**	0.792	2.169	.031
						Negativity						
Pleasant	**2.673**	**7.173**	**<.001**	**2.680**	**7.285**	**<.001**	**2.997**	**9.128**	**<.001**	**2.276**	**5.952**	**<.001**
Neutral	**1.459**	**3.751**	**<.001**	**1.833**	**4.843**	**<.001**	**2.099**	**6.071**	**<.001**	**1.458**	**3.749**	**<.001**
Unpleasant	**-1.253**	**-3.733**	**<.001**	**-0.958**	**-2.853**	**.005**	**-1.589**	**-5.244**	**<.001**	**-1.010**	**-2.983**	**.003**
						Arousal						
Pleasant	**2.451**	**5.941**	**<.001**	**2.951**	**7.412**	**<.001**	**3.273**	**9.093**	**<.001**	**1.385**	**3.236**	**.001**
Neutral	**1.513**	**3.959**	**<.001**	**2.245**	**6.130**	**<.001**	**2.572**	**7.731**	**<.001**	**1.015**	**2.623**	**.009**
Unpleasant	**-1.922**	**-3.041**	**.003**	**-1.549**	**-2.522**	**.012**	**-2.588**	**-4.854**	**<.001**	**-1.596**	**-2.453**	**.015**
						Ambivalence						
Pleasant	**3.77**	**6.44**	**<.001**	**3.81**	**6.60**	**<.001**	**4.24**	**8.12**	**<.001**	**3.73**	**6.39**	**<.001**
Neutral	**1.90**	**2.81**	**.005**	**2.36**	**3.51**	**.001**	**3.03**	**5.02**	**<.001**	**2.13**	**3.15**	**.002**
Unpleasant	**2.01**	**3.26**	**.001**	**1.75**	**2.87**	**.004**	**3.11**	**5.66**	**<.001**	**2.06**	**3.35**	**.001**

Note: N = 261. CAPE = Community Assessment of Psychic Experiences; CAS = Chapman Physical and Social Anhedonia Scales. Questionnaires were normalized to proportions (range 0-1) and centered to the respective sample mean. Scale range for Positivity, Negativity, Arousal = 0-8; for Ambivalence = 0-45. All effects with $p < .016$ were significant after Bonferroni correction for 3 tests (printed bold).

Positivity: Full models (Tables S4-S8)

Table S4. Results for the base positivity MLM (no moderator included).

	B	F	t	p	IC value
**Information criteria**					
-2 Log likelihood					47602.97
AIC					47626.97
**Tests of fixed effects**					
Intercept		492.26		<.001	
Category		342.94		<.001	
Gender		0.12		.729	
Age		3.80		.052	
Years of education		4.95		.027	
**Estimates of fixed effects**					
Intercept [Category = unpleasant]	1.37		6.37	<.001	
Category = pleasant	4.90		26.06	<.001	
Category = neutral	2.95		15.68	<.001	
Gender	0.03		0.35	.729	
Age	0.01		1.95	.052	
Years of education	-0.02		-2.22	.027	
	**Est.**		**Wald Z**	**p**	
**By-subject random variances**					
Intercept	0.16		3.01	.003	
Category = pleasant	0.65		7.57	<.001	
Category = neutral	0.64		6.18	<.001	
Category = unpleasant	0.96		8.67	<.001	
**By-stimulus random variances**					
Intercept	0.45		6.61	<.001	
**Residual variance**	2.29		76.19	<.001	

Note. B coefficients are unstandardized.

Table S5. Results for the positivity MLM with CAPE negative as moderator.

	B	F	t	p	IC value
**Information criteria**					
-2 Log likelihood					47555.02
AIC					47589.02
**Tests of fixed effects**					
Intercept		301.12		<.001	
Category		347.28		<.001	
Gender		0.03		.871	
Age		9.27		.003	
Years of education		1.20		.275	
CAPE depression		0.32		.574	
CAPE positive		23.35		<.001	
CAPE negative		7.75		.006	
Category*CAPE negative		11.63		<.001	
**Estimates of fixed effects**					
Intercept [Category = unpleasant]	1.06		4.36	<.001	
Category = pleasant	4.90		26.20	<.001	
Category = neutral	2.95		15.77	<.001	
Gender	-0.01		-0.16	.871	
Age	0.01		3.04	.003	
Years of education	-0.01		-1.09	.275	
CAPE depression	-0.21		-0.56	.574	
CAPE positive	1.68		4.83	<.001	
CAPE negative [Category = unpleasant]	0.14		0.31	.758	
CAPE negative [pleasant vs. unpleasant]	-2.11		-4.79	<.001	
CAPE negative [neutral vs. unpleasant]	-1.36		-3.09	.002	
	**Est.**		**Wald Z**	**p**	
**By-subject random variances**					
Intercept	0.11		2.34	.020	
Category = pleasant	0.68		7.90	<.001	
Category = neutral	0.66		6.39	<.001	
Category = unpleasant	0.83		8.69	<.001	
**By-stimulus random variances**					
Intercept	0.45		6.61	<.001	
**Residual variance**	2.29		76.19	<.001	

Note. B coefficients are unstandardized. CAPE = Community Assessment of Psychic Experiences normalized to proportions (0-1) and centered to sample mean.

Table S6. Results for the positivity MLM with CAPE depression as moderator.

	B	F	t	p	IC value
**Information criteria**					
-2 Log likelihood					47563.55
AIC					47597.55
**Tests of fixed effects**					
Intercept		299.64		<.001	
Category		345.87		<.001	
Gender		0.02		.883	
Age		9.28		.003	
Years of education		1.19		.278	
CAPE negative		8.81		.003	
CAPE positive		23.17		<.001	
CAPE depression		0.17		.679	
Category*CAPE depression		7.19		.001	
**Estimates of fixed effects**					
Intercept [Category = unpleasant]	1.06		4.34	<.001	
Category = pleasant	4.90		26.15	<.001	
Category = neutral	2.95		15.75	<.001	
Gender	-0.01		-0.15	.883	
Age	0.01		3.05	.003	
Years of education	-0.01		-1.09	.278	
CAPE negative	-1.08		-2.97	.003	
CAPE positive	1.68		4.81	<.001	
CAPE depression [Category = unpleasant]	0.74		1.58	.116	
CAPE depression [pleasant vs. unpleasant]	-1.67		-3.78	<.001	
CAPE depression [neutral vs. unpleasant]	-1.02		-2.33	.021	
	**Est.**		**Wald Z**	**p**	
**By-subject random variances**					
Intercept	0.11		2.26	.024	
Category = pleasant	0.69		7.88	<.001	
Category = neutral	0.66		6.36	<.001	
Category = unpleasant	0.86		8.70	<.001	
**By-stimulus random variances**					
Intercept	0.45		6.61	<.001	
**Residual variance**	2.29		76.19	<.001	

Note. B coefficients are unstandardized. CAPE = Community Assessment of Psychic Experiences normalized to proportions (0-1) and centered to sample mean.

Table S7. Results for the positivity MLM with CAPE positive as moderator.

	B	F	t	p	IC value
**Information criteria**					
-2 Log likelihood					47540.29
AIC					47574.29
**Tests of fixed effects**					
Intercept		302.41		<.001	
Category		350.13		<.001	
Gender		0.02		.890	
Age		9.20		.003	
Years of education		1.14		.286	
CAPE depression		0.30		.586	
CAPE negative		8.89		.003	
CAPE positive		25.32		<.001	
Category*CAPE positive		19.48		<.001	
**Estimates of fixed effects**					
Intercept [Category = unpleasant]	1.06		4.37	<.001	
Category = pleasant	4.90		26.31	<.001	
Category = neutral	2.95		15.82	<.001	
Gender	-0.01		-0.14	.890	
Age	0.01		3.03	.003	
Years of education	-0.01		-1.07	.286	
CAPE depression	-0.21		-0.55	.586	
CAPE negative	-1.08		-2.98	.003	
CAPE positive [Category = unpleasant]	3.01		7.02	<.001	
CAPE positive [pleasant vs. unpleasant]	-2.51		-6.23	<.001	
CAPE positive [neutral vs. unpleasant]	-1.27		-3.14	.002	
	**Est.**		**Wald Z**	**p**	
**By-subject random variances**					
Intercept	0.11		2.49	.013	
Category = pleasant	0.64		7.81	<.001	
Category = neutral	0.65		6.40	<.001	
Category = unpleasant	0.82		8.68	<.001	
**By-stimulus random variances**					
Intercept	0.45		6.62	<.001	
**Residual variance**	2.29		76.19	<.001	

Note. B coefficients are unstandardized. CAPE = Community Assessment of Psychic Experiences normalized to proportions (0-1) and centered to sample mean.

Table S8. Results for the positivity MLM with CAS anhedonia as moderator.

	B	F	t	p	IC value
**Information criteria**					
-2 Log likelihood					47549.64
AIC					47583.64
**Tests of fixed effects**					
Intercept		306.48		<.001	
Category		346.48		<.001	
Gender		0.00		.965	
Age		8.57		.004	
Years of education		2.31		.130	
CAPE depression		3.79		.053	
CAPE positive		20.28		<.001	
CAS anhedonia		9.14		.003	
Category*CAS anhedonia		13.19		<.001	
**Estimates of fixed effects**					
Intercept [Category = unpleasant]	1.12		4.59	<.001	
Category = pleasant	4.90		26.18	<.001	
Category = neutral	2.95		15.74	<.001	
Gender	0.00		0.04	.965	
Age	0.01		2.93	.004	
Years of education	-0.01		-1.52	.130	
CAPE depression	-0.62		-1.95	.053	
CAPE positive	1.53		4.50	<.001	
CAS anhedonia [Category = unpleasant]	0.50		1.36	.175	
CAS anhedonia [pleasant vs. unpleasant]	-2.24		-5.10	<.001	
CAS anhedonia [neutral vs. unpleasant]	-1.46		-3.33	.001	
	**Est.**		**Wald Z**	**p**	
**By-subject random variances**					
Intercept	0.11		2.34	.020	
Category = pleasant	0.66		7.84	<.001	
Category = neutral	0.66		6.41	<.001	
Category = unpleasant	0.84		8.71	<.001	
**By-stimulus random variances**					
Intercept	0.45		6.61	<.001	
**Residual variance**	2.29		76.19	<.001	

Note. B coefficients are unstandardized. CAS = Chapman Anhedonia Scales normalized to proportions (0-1) and centered to sample mean.

Negativity: Full models (Tables S9-S13)

Table S9. Results for the base negativity MLM (no moderator included).

	B	F	t	p	IC value
**Information criteria**					
-2 Log likelihood					49501.96
AIC					49525.96
**Tests of fixed effects**					
Intercept		499.18		<.001	
Category		327.66		<.001	
Gender		4.82		.028	
Age		1.99		.159	
Years of education		8.39		.004	
**Estimates of fixed effects**					
Intercept [Category = unpleasant]	6.72		31.42	<.001	
Category = pleasant	-4.84		-24.62	<.001	
Category = neutral	-3.55		-18.11	<.001	
Gender	0.19		2.20	.028	
Age	0.00		-1.41	.159	
Years of education	-0.02		-2.90	.004	
	**Est.**		**Wald Z**	**p**	
**By-subject random variances**					
Intercept	-		-	-	
Category = pleasant	1.29		9.69	<.001	
Category = neutral	1.24		9.93	<.001	
Category = unpleasant	1.00		9.01	<.001	
**By-stimulus random variances**					
Intercept	0.46		6.57	<.001	
**Residual variance**	2.65		76.19	<.001	

Note. B coefficients are unstandardized.

Table S10. Results for the negativity MLM with CAPE negative as moderator.

	B	F	t	p	IC value
**Information criteria**					
-2 Log likelihood					49418.38
AIC					49452.38
**Tests of fixed effects**					
Intercept		277.22		<.001	
Category		335.91		<.001	
Gender		5.15		.023	
Age		0.76		.382	
Years of education		2.04		.154	
CAPE depression		2.14		.144	
CAPE positive		4.41		.036	
CAPE negative		0.01		.912	
Category*CAPE negative		33.41		<.001	
**Estimates of fixed effects**					
Intercept [Category = unpleasant]	6.24		25.53	<.001	
Category = pleasant	-4.84		-25.01	<.001	
Category = neutral	-3.55		-18.25	<.001	
Gender	0.19		2.27	.023	
Age	0.00		0.87	.382	
Years of education	-0.01		-1.43	.154	
CAPE depression	0.56		1.46	.144	
CAPE positive	0.73		2.10	.036	
CAPE negative [Category = unpleasant]	-2.20		-4.78	<.001	
CAPE negative [pleasant vs. unpleasant]	3.98		7.97	<.001	
CAPE negative [neutral vs. unpleasant]	2.73		5.35	<.001	
	**Est.**		**Wald Z**	**p**	
**By-subject random variances**					
Intercept	-		-	-	
Category = pleasant	1.03		9.49	<.001	
Category = neutral	1.14		9.81	<.001	
Category = unpleasant	0.94		9.00	<.001	
**By-stimulus random variances**					
Intercept	0.46		6.58	<.001	
**Residual variance**	2.65		76.19	<.001	

Note. B coefficients are unstandardized. CAPE = Community Assessment of Psychic Experiences normalized to proportions (0-1) and centered to sample mean.

Table S11. Results for the negativity MLM with CAPE depression as moderator.

	B	F	t	p	IC value
**Information criteria**					
-2 Log likelihood					49423.71
AIC					49457.71
**Tests of fixed effects**					
Intercept		275.37		<.001	
Category		334.91		<.001	
Gender		5.22		.023	
Age		0.76		.385	
Years of education		2.00		.158	
CAPE negative		0.01		.910	
CAPE positive		4.33		.038	
CAPE depression		2.84		.092	
Category*CAPE depression		30.42		<.001	
**Estimates of fixed effects**					
Intercept [Category = unpleasant]	6.24		25.45	<.001	
Category = pleasant	-4.84		-24.96	<.001	
Category = neutral	-3.55		-18.24	<.001	
Gender	0.20		2.28	.023	
Age	0.00		0.87	.385	
Years of education	-0.01		-1.41	.158	
CAPE negative	-0.04		-0.11	.910	
CAPE positive	0.73		2.08	.038	
CAPE depression [Category = unpleasant]	-1.53		-3.25	.001	
CAPE depression [pleasant vs. unpleasant]	3.70		7.43	<.001	
CAPE depression [neutral vs. unpleasant]	2.82		5.57	<.001	
	**Est.**		**Wald Z**	**p**	
**By-subject random variances**					
Intercept	-		-	-	
Category = pleasant	1.06		9.46	<.001	
Category = neutral	1.13		9.82	<.001	
Category = unpleasant	0.94		8.99	<.001	
**By-stimulus random variances**					
Intercept	0.46		6.58	<.001	
**Residual variance**	2.65		76.19	<.001	

Note. B coefficients are unstandardized. CAPE = Community Assessment of Psychic Experiences normalized to proportions (0-1) and centered to sample mean.

Table S12. Results for the negativity MLM with CAPE positive as moderator.

	B	F	t	p	IC value
**Information criteria**					
-2 Log likelihood					49367.76
AIC					49401.76
**Tests of fixed effects**					
Intercept		291.91		<.001	
Category		344.70		<.001	
Gender		6.24		.013	
Age		0.83		.363	
Years of education		1.72		.190	
CAPE depression		2.37		.124	
CAPE negative		0.01		.914	
CAPE positive		6.29		.012	
Category*CAPE positive		63.17		<.001	
**Estimates of fixed effects**					
Intercept [Category = unpleasant]	6.21		26.18	<.001	
Category = pleasant	-4.84		-25.30	<.001	
Category = neutral	-3.55		-18.45	<.001	
Gender	0.21		2.50	.013	
Age	0.00		0.91	.363	
Years of education	-0.01		-1.31	.190	
CAPE depression	0.57		1.54	.124	
CAPE negative	-0.04		-0.11	.914	
CAPE positive [Category = unpleasant]	-1.96		-4.76	<.001	
CAPE positive [pleasant vs. unpleasant]	4.69		10.53	<.001	
CAPE positive [neutral vs. unpleasant]	3.74		8.18	<.001	
	**Est.**		**Wald Z**	**p**	
**By-subject random variances**					
Intercept	-		-	-	
Category = pleasant	0.99		9.59	<.001	
Category = neutral	1.11		9.87	<.001	
Category = unpleasant	0.80		9.22	<.001	
**By-stimulus random variances**					
Intercept	0.45		6.58	<.001	
**Residual variance**	2.65		76.19	<.001	

Note. B coefficients are unstandardized. Model needs to be compared to the MLM in Table S6, $\chi^2(3)=74.16$, $p<.001$. CAPE = Community Assessment of Psychic Experiences normalized to proportions (0-1) and centered to sample mean.

Table S13. Results for the negativity MLM with CAS anhedonia as moderator.

	B	F	t	p	IC value
**Information criteria**					
-2 Log likelihood					49432.77
AIC					49466.77
**Tests of fixed effects**					
Intercept		253.45		<.001	
Category		332.04		<.001	
Gender		6.25		.013	
Age		1.48		.224	
Years of education		1.41		.235	
CAPE depression		1.67		.197	
CAPE positive		4.36		.037	
CAS anhedonia		3.83		.051	
Category*CAS anhedonia		23.69		<.001	
**Estimates of fixed effects**					
Intercept [Category = unpleasant]	6.16		24.68	<.001	
Category = pleasant	-4.84		-24.87	<.001	
Category = neutral	-3.55		-18.18	<.001	
Gender	0.21		2.50	.013	
Age	0.00		1.22	.224	
Years of education	-0.01		-1.19	.235	
CAPE depression	0.42		1.29	.197	
CAPE positive	0.72		2.09	.037	
CAS anhedonia [Category = unpleasant]	-1.47		-3.88	<.001	
CAS anhedonia [pleasant vs. unpleasant]	3.36		6.61	<.001	
CAS anhedonia [neutral vs. unpleasant]	2.50		4.85	<.001	
	**Est.**		**Wald Z**	**p**	
**By-subject random variances**					
Intercept	-		-	-	
Category = pleasant	1.05		9.39	<.001	
Category = neutral	1.13		9.81	<.001	
Category = unpleasant	0.99		8.93	<.001	
**By-stimulus random variances**					
Intercept	0.46		6.57	<.001	
**Residual variance**	2.65		76.19	<.001	

Note. B coefficients are unstandardized. CAS = Physical and Social Anhedonia Scale normalized to proportions (0-1) and centered to sample mean.

Arousal: Full models (Tables S14-S18)

Table S14. Results for the base arousal MLM (no moderator included).

	B	F	t	p	IC value
**Information criteria**					
-2 Log likelihood					49456.85
AIC					49480.85
**Tests of fixed effects**					
Intercept		411.46		<.001	
Category		49.97		<.001	
Gender		0.00		.994	
Age		20.49		<.001	
Years of education		9.54		.002	
**Estimates of fixed effects**					
Intercept [Category = unpleasant]	6.45		21.10	<.001	
Category = pleasant	-1.22		-5.60	<.001	
Category = neutral	-2.15		-9.97	<.001	
Gender	0.00		0.01	.994	
Age	-0.02		-4.53	<.001	
Years of education	-0.04		-3.09	.002	
	**Est.**		**Wald Z**	**p**	
**By-subject random variances**					
Intercept	0.68		6.58	<.001	
Category = pleasant	0.73		6.57	<.001	
Category = neutral	0.45		4.64	<.001	
Category = unpleasant	3.46		9.77	<.001	
**By-stimulus random variances**					
Intercept	0.48		6.61	<.001	
**Residual variance**	2.59		76.19	<.001	

Note. B coefficients are unstandardized.

Table S15. Results for the arousal MLM with CAPE negative as moderator.

	B	F	t	p	IC value
**Information criteria**					
-2 Log likelihood					49382.97
AIC					49416.97
**Tests of fixed effects**					
Intercept		208.73		<.001	
Category		51.32		<.001	
Gender		0.05		.820	
Age		2.84		.093	
Years of education		1.03		.310	
CAPE depression		6.18		.014	
CAPE positive		11.92		.001	
CAPE negative		15.85		<.001	
Category*CAPE negative		20.94		<.001	
**Estimates of fixed effects**					
Intercept [Category = unpleasant]	5.58		16.10	<.001	
Category = pleasant	-1.22		-5.68	<.001	
Category = neutral	-2.15		-10.12	<.001	
Gender	-0.03		-0.23	.820	
Age	-0.01		-1.69	.093	
Years of education	-0.01		-1.02	.310	
CAPE depression	1.40		2.49	.014	
CAPE positive	1.79		3.45	.001	
CAPE negative [Category = unpleasant]	-4.80		-6.14	<.001	
CAPE negative [pleasant vs. unpleasant]	4.29		6.47	<.001	
CAPE negative [neutral vs. unpleasant]	3.39		5.24	<.001	
	**Est.**		**Wald Z**	**p**	
**By-subject random variances**					
Intercept	0.56		6.26	<.001	
Category = pleasant	0.66		6.47	<.001	
Category = neutral	0.46		5.00	<.001	
Category = unpleasant	3.08		9.78	<.001	
**By-stimulus random variances**					
Intercept	0.48		6.61	<.001	
**Residual variance**	2.59		76.19	<.001	

Note. B coefficients are unstandardized. CAPE = Community Assessment of Psychic Experiences normalized to proportions (0-1) and centered to sample mean.

Table S16. Results for the arousal MLM with CAPE depression as moderator.

	B	F	t	p	IC value
**Information criteria**					
-2 Log likelihood					49379.30
AIC					49413.30
**Tests of fixed effects**					
Intercept		209.05		<.001	
Category		51.62		<.001	
Gender		0.04		.841	
Age		2.76		.098	
Years of education		1.02		.313	
CAPE negative		6.58		.011	
CAPE positive		11.75		.001	
CAPE depression		0.74		.390	
Category*CAPE depression		23.19		<.001	
**Estimates of fixed effects**					
Intercept [Category = unpleasant]	5.57		16.12	<.001	
Category = pleasant	-1.22		-5.70	<.001	
Category = neutral	-2.15		-10.15	<.001	
Gender	-0.03		-0.20	.841	
Age	-0.01		-1.66	.098	
Years of education	-0.01		-1.01	.313	
CAPE negative	-1.39		-2.57	.011	
CAPE positive	1.77		3.43	.001	
CAPE depression [Category = unpleasant]	-2.22		-2.82	.005	
CAPE depression [pleasant vs. unpleasant]	4.41		6.77	<.001	
CAPE depression [neutral vs. unpleasant]	3.75		5.91	<.001	
	**Est.**		**Wald Z**	**p**	
**By-subject random variances**					
Intercept	0.55		6.19	<.001	
Category = pleasant	0.68		6.53	<.001	
Category = neutral	0.46		5.00	<.001	
Category = unpleasant	2.99		9.74	<.001	
**By-stimulus random variances**					
Intercept	0.48		6.61	<.001	
**Residual variance**	2.59		76.19	<.001	

Note. B coefficients are unstandardized. CAPE = Community Assessment of Psychic Experiences normalized to proportions (0-1) and centered to sample mean.

Table S17. Results for the arousal MLM with CAPE positive as moderator.

	B	F	t	p	IC value
**Information criteria**					
-2 Log likelihood					49328.79
AIC					49362.79
**Tests of fixed effects**					
Intercept		211.36		<.001	
Category		54.18		<.001	
Gender		0.01		.922	
Age		2.46		.118	
Years of education		0.82		.366	
CAPE depression		6.33		.012	
CAPE negative		6.42		.012	
CAPE positive		1.18		.278	
Category*CAPE positive		55.18		<.001	
**Estimates of fixed effects**					
Intercept [Category = unpleasant]	5.52		16.32	<.001	
Category = pleasant	-1.21		-5.84	<.001	
Category = neutral	-2.15		-10.41	<.001	
Gender	-0.01		-0.10	.922	
Age	-0.01		-1.57	.118	
Years of education	-0.01		-0.91	.366	
CAPE depression	1.39		2.52	.012	
CAPE negative	-1.35		-2.53	.012	
CAPE positive [Category = unpleasant]	-3.08		-4.49	<.001	
CAPE positive [pleasant vs. unpleasant]	5.79		10.26	<.001	
CAPE positive [neutral vs. unpleasant]	5.14		9.39	<.001	
	**Est.**		**Wald Z**	**p**	
**By-subject random variances**					
Intercept	0.53		6.08	<.001	
Category = pleasant	0.68		6.60	<.001	
Category = neutral	0.48		5.16	<.001	
Category = unpleasant	2.40		9.52	<.001	
**By-stimulus random variances**					
Intercept	0.48		6.62	<.001	
**Residual variance**	2.59		76.19	<.001	

Note. B coefficients are unstandardized. CAPE = Community Assessment of Psychic Experiences normalized to proportions (0-1) and centered to sample mean.

Table S18. Results for the arousal MLM with CAS anhedonia as moderator.

	B	F	t	p	IC value
**Information criteria**					
-2 Log likelihood					49409.09
AIC					49443.09
**Tests of fixed effects**					
Intercept		199.52		<.001	
Category		50.40		<.001	
Gender		0.01		.924	
Age		2.31		.130	
Years of education		1.49		.223	
CAPE depression		2.34		.127	
CAPE positive		9.56		.002	
CAS anhedonia		9.05		.003	
Category*CAS anhedonia		9.06		<.001	
**Estimates of fixed effects**					
Intercept [Category = unpleasant]	5.58		15.70	<.001	
Category = pleasant	-1.22		-5.63	<.001	
Category = neutral	-2.15		-10.02	<.001	
Gender	0.01		0.10	.924	
Age	-0.01		-1.52	.130	
Years of education	-0.02		-1.22	.223	
CAPE depression	0.74		1.53	.127	
CAPE positive	1.59		3.09	.002	
CAS anhedonia [Category = unpleasant]	-3.01		-4.37	<.001	
CAS anhedonia [pleasant vs. unpleasant]	2.88		4.21	<.001	
CAS anhedonia [neutral vs. unpleasant]	2.55		3.83	<.001	
	**Est.**		**Wald Z**	**p**	
**By-subject random variances**					
Intercept	0.57		6.25	<.001	
Category = pleasant	0.68		6.52	<.001	
Category = neutral	0.46		4.93	<.001	
Category = unpleasant	3.30		9.83	<.001	
**By-stimulus random variances**					
Intercept	0.48		6.61	<.001	
**Residual variance**	2.59		76.19	<.001	

Note. B coefficients are unstandardized. CAS = Chapman Physical and Social Anhedonia Scale normalized to proportions (0-1) and centered to sample mean.

Ambivalence: Full models (Tables S19-S23)

Table S19. Results for the base ambivalence MLM (no moderator included).

	B	F	t	p	IC value
**Information criteria**					
-2 Log likelihood					98680.59
AIC					98704.59
**Tests of fixed effects**					
Intercept		147.20		<.001	
Category		61.39		<.001	
Gender		3.97		.047	
Age		10.26		.002	
Years of education		18.63		<.001	
**Estimates of fixed effects**					
Intercept [Category = unpleasant]	24.72		10.15	<.001	
Category = pleasant	0.90		0.80	.424	
Category = neutral	12.25		10.09	<.001	
Gender	-2.38		-1.99	.047	
Age	-0.13		-3.20	.002	
Years of education	-0.49		-4.32	<.001	
	**Est.**		**Wald Z**	**p**	
**By-subject random variances**					
Intercept	72.23		9.58	<.001	
Category = pleasant	8.66		2.54	.011	
Category = neutral	64.68		8.55	<.001	
Category = unpleasant	20.65		5.10	<.001	
**By-stimulus random variances**					
Intercept	17.10		6.38	<.001	
**Residual variance**	157.22		75.52	<.001	

Note. B coefficients are unstandardized.

Table S20. Results for the ambivalence MLM with CAPE negative as moderator.

	B	F	t	p	IC value
**Information criteria**					
-2 Log likelihood					56342.96
AIC					56376.96
**Tests of fixed effects**					
Intercept		.10		.751	
Category		70.48		<.001	
Gender		4.16		.043	
Age		0.27		.606	
Years of education		6.42		.012	
CAPE depression		0.02		.899	
CAPE positive		12.57		<.001	
CAPE negative		0.18		.671	
Category*CAPE negative		12.70		<.001	
**Estimates of fixed effects**					
Intercept [Category = unpleasant]	-0.52		-0.99	.326	
Category = pleasant	0.31		1.49	.139	
Category = neutral	2.39		10.99	<.001	
Gender	-0.43		-2.04	.043	
Age	-0.00		-0.52	.606	
Years of education	-0.05		-2.53	.012	
CAPE depression	-0.12		-0.13	.899	
CAPE positive	3.07		3.55	<.001	
CAPE negative [Category = unpleasant]	-0.97		-1.02	.309	
CAPE negative [pleasant vs. unpleasant]	1.86		4.35	<.001	
CAPE negative [neutral vs. unpleasant]	-0.12		-0.22	.829	
	**Est.**		**Wald Z**	**p**	
**By-subject random variances**					
Intercept	2.12		9.42	<.001	
Category = pleasant	0.39		3.58	<.001	
Category = neutral	1.44		7.67	<.001	
Category = unpleasant	0.71		5.54	<.001	
**By-stimulus random variances**					
Intercept	0.59		6.46	<.001	
**Residual variance**	4.63		76.19	<.001	

Note. B coefficients are unstandardized. CAPE = Community Assessment of Psychic Experiences normalized to proportions (0-1) and centered to sample mean.

Table S21. Results for the ambivalence MLM with CAPE depression as moderator.

	B	F	t	p	IC value
**Information criteria**					
-2 Log likelihood					56342.59
AIC					56376.59
**Tests of fixed effects**					
Intercept		0.10		.751	
Category		70.23		<.001	
Gender		4.31		.039	
Age		0.27		.605	
Years of education		6.43		.012	
CAPE negative		0.01		.913	
CAPE positive		12.68		<.001	
CAPE depression		0.13		.714	
Category*CAPE depression		13.31		<.001	
**Estimates of fixed effects**					
Intercept [Category = unpleasant]	-0.52		0.98	.328	
Category = pleasant	0.31		1.50	.137	
Category = neutral	2.39		10.99	<.001	
Gender	-0.44		-2.08	.039	
Age	-0.004		-0.52	.605	
Years of education	-0.05		-2.54	.012	
CAPE negative	-0.10		-0.11	.913	
CAPE positive	3.08		3.56	<.001	
CAPE depression [Category = unpleasant]	-1.24		-1.27	.206	
CAPE depression [pleasant vs. unpleasant]	2.07		4.95	<.001	
CAPE depression [neutral vs. unpleasant]	0.61		1.13	.258	
	**Est.**		**Wald Z**	**p**	
**By-subject random variances**					
Intercept	2.12		9.45	<.001	
Category = pleasant	0.40		3.65	<.001	
Category = neutral	1.49		7.76	<.001	
Category = unpleasant	0.66		5.35	<.001	
**By-stimulus random variances**					
Intercept	0.59		6.46	<.001	
**Residual variance**	4.63		76.19	<.001	

Note. B coefficients are unstandardized. CAPE = Community Assessment of Psychic Experiences normalized to proportions (0-1) and centered to sample mean.

Table S22. Results for the ambivalence MLM with CAPE positive as moderator.

	B	F	t	p	IC value
**Information criteria**					
-2 Log likelihood					56356.94
AIC					56390.94
**Tests of fixed effects**					
Intercept		0.10		.752	
Category		70.30		<.001	
Gender		4.18		.042	
Age		0.28		.600	
Years of education		6.40		.012	
CAPE depression		0.02		.881	
CAPE negative		0.02		.893	
CAPE positive		11.42		<.001	
Category*CAPE positive		5.26		.006	
**Estimates of fixed effects**					
Intercept [Category = unpleasant]	-0.53		0.99	.322	
Category = pleasant	0.31		1.49	.140	
Category = neutral	2.39		10.98	<.001	
Gender	-0.43		-2.05	.042	
Age	-0.004		-0.53	.600	
Years of education	-0.05		-2.53	.012	
CAPE depression	-0.14		-0.15	.881	
CAPE negative	-0.12		-0.14	.893	
CAPE positive [Category = unpleasant]	2.57		2.86	.005	
CAPE positive [pleasant vs. unpleasant]	1.13		2.80	.006	
CAPE positive [neutral vs. unpleasant]	-0.08		-0.16	.875	
	**Est.**		**Wald Z**	**p**	
**By-subject random variances**					
Intercept	2.08		9.37	<.001	
Category = pleasant	0.45		3.92	<.001	
Category = neutral	1.45		7.62	<.001	
Category = unpleasant	0.73		5.53	<.001	
**By-stimulus random variances**					
Intercept	0.59		6.46	<.001	
**Residual variance**	4.63		76.19	<.001	

Note. B coefficients are unstandardized. CAPE = Community Assessment of Psychic Experiences normalized to proportions (0-1) and centered to sample mean.

Table S23. Results for the ambivalence MLM with CAS anhedonia as moderator.

	B	F	t	p	IC value
**Information criteria**					
-2 Log likelihood					56345.78
AIC					56379.78
**Tests of fixed effects**					
Intercept		.01		.919	
Category		70.19		<.001	
Gender		3.22		.074	
Age		0.02		.888	
Years of education		5.00		.026	
CAPE depression		0.34		.561	
CAPE positive		12.23		.001	
CAS anhedonia		2.25		.135	
Category*CAS anhedonia		9.34		<.001	
**Estimates of fixed effects**					
Intercept [Category = unpleasant]	-0.77		-1.43	.154	
Category = pleasant	0.31		1.49	.139	
Category = neutral	2.39		10.97	<.001	
Gender	-0.37		-1.80	.074	
Age	-0.001		-0.14	.888	
Years of education	-0.05		-2.24	.026	
CAPE depression	-0.46		-0.58	.561	
CAPE positive	2.95		3.50	<.001	
CAS anhedonia [Category = unpleasant]	0.33		0.51	.614	
CAS anhedonia [pleasant vs. unpleasant]	1.67		3.88	<.001	
CAS anhedonia [neutral vs. unpleasant]	0.06		0.12	.906	
	**Est.**		**Wald Z**	**p**	
**By-subject random variances**					
Intercept	2.08		9.39	<.001	
Category = pleasant	0.40		3.64	<.001	
Category = neutral	1.46		7.70	<.001	
Category = unpleasant	0.72		5.58	<.001	
**By-stimulus random variances**					
Intercept	0.59		6.45	<.001	
**Residual variance**	4.63		76.19	<.001	

Note. B coefficients are unstandardized. CAS = Chapman Physical and Social Anhedonia Scale normalized to proportions (0-1) and centered to sample mean.

Positivity offset analyses

Full positivity offset models (Tables S24-S28)

Table S24. Results for the positivity offset base MLM with no moderator included.

	B	F	t	p	IC value
**Information criteria**					
-2 Log likelihood					48705.54
AIC					48729.54
**Tests of fixed effects**					
Intercept		383.16		<.001	
Gender		5.98		.015	
Age		2.68		.102	
Years of education		8.69		.003	
Activation function		84.30		<.001	
Arousal		261.24		<.001	
Activation function*arousal		190.64		<.001	
**Estimates of fixed effects**					
Intercept [activation function = positivity]	4.61		20.67	<.001	
Activation function = negativity	-2.37		-9.18	<.001	
Gender	0.16		2.45	.015	
Age	0.00		1.64	.102	
Years of education	0.02		2.95	.003	
Arousal [activation function = positivity]	0.03		1.64	.101	
Arousal [negativity vs. positivity]	0.40		13.81	<.001	
	**Est.**		**Wald Z**	**p**	
**By-subject random variances**					
Intercept	1.96		12.05	<.001	
Arousal	0.07		11.19	<.001	
Intercept*Arousal (Cov)	-0.33		-11.04	<.001	
**By-stimulus random variances**					
Intercept	1.48		7.78	<.001	
**Residual variance**	2.48		75.19	<.001	

Note. B coefficients are unstandardized.

Table S25. Results for the positivity offset base MLM with CAPE negative as moderator.

	B	F	t	p	IC value
**Information criteria**					
-2 Log likelihood					48705.54
AIC					48729.54
**Tests of fixed effects**					
Intercept		318.38		<.001	
Gender		4.08		.044	
Age		0.35		.556	
Years of education		6.05		.014	
Activation function		87.00		<.001	
Arousal		274.25		<.001	
Activation function*arousal		203.83		<.001	
CAPE depression		0.18		.674	
CAPE positive		0.00		.986	
CAPE negative		1.41		.236	
Activation function*CAPE negative		32.83		<.001	
Arousal*CAPE negative		0.05		.817	
Activation function*Arousal *CAPE negative		32.01		<.001	
**Estimates of fixed effects**					
Intercept [activation function = positivity]	4.74		19.52	<.001	
Activation function = negativity	-2.39		-9.33	<.001	
Gender	0.14		2.02	.044	
Age	0.00		0.59	.556	
Years of education	0.02		2.46	.014	
Arousal [activation function = positivity]	0.03		1.60	.111	
Arousal [negativity vs. positivity]	0.41		14.28	<.001	
CAPE depression	0.13		0.42	.674	
CAPE positive	0.00		-0.02	.986	
CAPE negative [activation function = positivity]	-2.59		-4.61	<.001	
CAPE negative [negativity vs. positivity]	4.15		5.73	<.001	
Arousal*CAPE negative [activation function = positivity]	0.42		4.10	<.001	
Arousal*CAPE negative [negativity vs. positivity]	-0.82		-5.66	<.001	

	Est.	Wald Z	p
**By-subject random variances**			
Intercept	1.82	11.99	<.001
Arousal	0.07	11.13	<.001
Intercept*Arousal (Cov)	-0.31	-10.95	<.001
**By-stimulus random variances**			
Intercept	1.47	11.13	<.001
**Residual variance**	2.47	75.22	<.001

Note. B coefficients are unstandardized. CAPE = Community Assessment of Psychic Experiences normalized to proportions (0-1) and centered to sample mean.

Table S26. Results for the positivity offset base MLM with CAPE depression as moderator.

	B	F	t	p	IC value
**Information criteria**					
-2 Log likelihood					48705.54
AIC					48729.54
**Tests of fixed effects**					
Intercept		317.15		<.001	
Gender		4.01		.046	
Age		0.36		.547	
Years of education		6.01		.015	
Activation function		86.09		<.001	
Arousal		275.77		<.001	
Activation function*arousal		201.72		<.001	
CAPE negative		2.56		.111	
CAPE positive		0.00		.982	
CAPE depression		0.42		.516	
Activation function*CAPE depression		28.41		<.001	
Arousal*CAPE depression		0.17		.681	
Activation function*Arousal *CAPE depression		30.67		<.001	
**Estimates of fixed effects**					
Intercept [activation function = positivity]	4.73		19.46	<.001	
Activation function = negativity	-2.38		-9.28	<.001	
Gender	0.14		2.00	.046	
Age	0.00		0.60	.547	
Years of education	0.02		2.45	.015	
Arousal [activation function = positivity]	0.03		1.68	.095	
Arousal [negativity vs. positivity]	0.41		14.20	<.001	
CAPE negative	-0.47		-1.60	.111	
CAPE positive	-0.01		-0.02	.982	
CAPE depression [activation function = positivity]	-1.64		-2.87	.004	
CAPE depression [negativity vs. positivity]	3.86		5.33	<.001	
Arousal*CAPE depression [activation function = positivity]	0.36		3.58	<.001	
Arousal*CAPE depression [negativity vs. positivity]	-0.79		-5.54	<.001	

	Est.	Wald Z	p
**By-subject random variances**			
Intercept	1.85	12.05	<.001
Arousal	0.07	11.12	<.001
Intercept*Arousal (Cov)	-0.31	-10.99	<.001
**By-stimulus random variances**			
Intercept	1.46	11.12	<.001
**Residual variance**	2.47	75.22	<.001

Note. B coefficients are unstandardized. CAPE = Community Assessment of Psychic Experiences normalized to proportions (0-1) and centered to sample mean.

Table S27. Results for the positivity offset base MLM with CAPE positive as moderator.

	B	F	t	p	IC value
**Information criteria**					
-2 Log likelihood					48705.54
AIC					48729.54
**Tests of fixed effects**					
Intercept		317.47		<.001	
Gender		3.66		.056	
Age		0.38		.536	
Years of education		6.36		.012	
Activation function		89.28		<.001	
Arousal		299.36		<.001	
Activation function*arousal		223.42		<.001	
CAPE negative		2.62		.106	
CAPE depression		0.15		.700	
CAPE positive		0.85		.356	
Activation function*CAPE positive		58.08		<.001	
Arousal*CAPE positive		1.33		.249	
Activation function*Arousal *CAPE positive		79.56		<.001	
**Estimates of fixed effects**					
Intercept [activation function = positivity]	4.72		19.58	<.001	
Activation function = negativity	-2.39		-9.45	<.001	
Gender	0.13		1.91	.056	
Age	0.00		0.62	.536	
Years of education	0.02		2.52	.012	
Arousal [activation function = positivity]	0.03		1.65	.100	
Arousal [negativity vs. positivity]	0.41		14.95	<.001	
CAPE negative	-0.48		-1.62	.106	
CAPE depression	0.12		0.39	.000	
CAPE positive [activation function = positivity]	-2.19		-4.10	<.001	
CAPE positive [negativity vs. positivity]	5.13		7.62	<.001	
Arousal*CAPE positive [activation function = positivity]	0.50		5.43	<.001	
Arousal*CAPE positive [negativity vs. positivity]	-1.16		-8.92	<.001	

	Est.	Wald Z	p
**By-subject random variances**			
Intercept	1.74	11.97	<.001
Arousal	0.06	10.87	<.001
Intercept*Arousal (Cov)	-0.28	-10.78	<.001
**By-stimulus random variances**			
Intercept	1.44	10.87	<.001
**Residual variance**	2.47	75.25	<.001

Note. B coefficients are unstandardized. CAPE = Community Assessment of Psychic Experiences normalized to proportions (0-1) and centered to sample mean.

Table S28. Results for the positivity offset base MLM with CAS anhedonia as moderator.

	B	F	t	p	IC value
**Information criteria**					
-2 Log likelihood					48705.54
AIC					48729.54
**Tests of fixed effects**					
Intercept		326.14		<.001	
Gender		3.92		.048	
Age		0.11		.739	
Years of education		3.58		.059	
Activation function		87.41		<.001	
Arousal		278.37		<.001	
Activation function*arousal		202.81		<.001	
CAPE depression		0.00		.960	
CAPE positive		0.01		.921	
CAS anhedonia		0.28		.598	
Activation function*CAS anhedonia		36.58		<.001	
Arousal*CAS anhedonia		5.15		.024	
Activation function*Arousal *CAS anhedonia		27.08		<.001	
**Estimates of fixed effects**					
Intercept [activation function = positivity]	4.82		19.72	<.001	
Activation function = negativity	-2.39		-9.35	<.001	
Gender	0.13		1.98	.048	
Age	0.00		0.33	.739	
Years of education	0.01		1.89	.059	
Arousal [activation function = positivity]	0.03		1.71	.088	
Arousal [negativity vs. positivity]	0.41		14.24	<.001	
CAPE depression	-0.01		-0.05	.960	
CAPE positive	-0.03		-0.10	.921	
CAS anhedonia [activation function = positivity]	-1.99		-3.88	<.001	
CAS anhedonia [negativity vs. positivity]	4.37		6.05	<.001	
Arousal*CAS anhedonia [activation function = positivity]	0.21		2.06	.040	
Arousal*CAS anhedonia [negativity vs. positivity]	-0.75		-5.20	<.001	

	Est.	Wald Z	p
**By-subject random variances**			
Intercept	1.80	11.94	<.001
Arousal	0.07	11.14	<.001
Intercept*Arousal (Cov)	-0.31	-10.91	<.001
**By-stimulus random variances**			
Intercept	1.47	11.14	<.001
**Residual variance**	2.47	75.23	<.001

Note. B coefficients are unstandardized. CAS = Chapman Physical and Social Anhedonia Scale normalized to proportions (0-1) and centered to sample mean.

Supplementary positivity offset figures (Figures S1-S3)

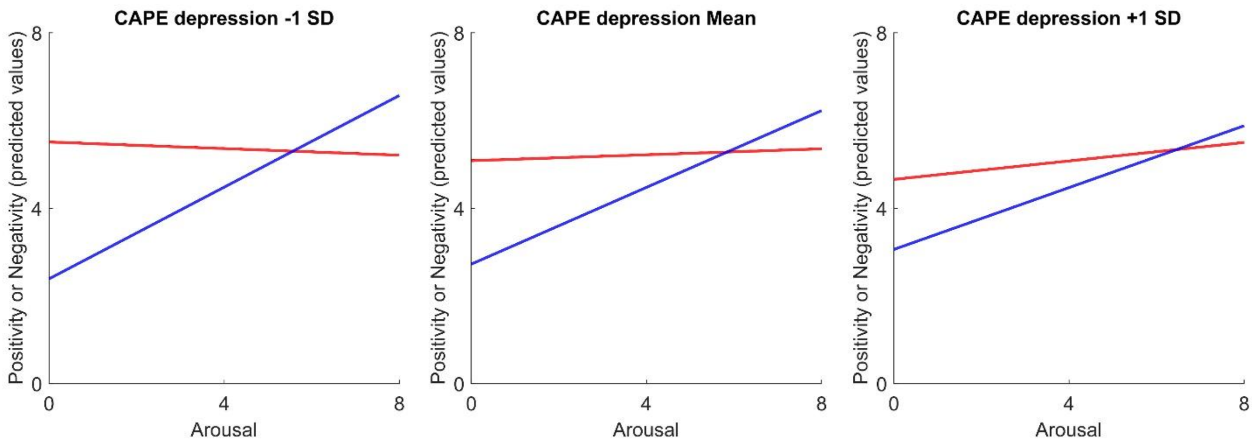

Figure S1. Positivity (red) and negativity (blue) activation functions estimated for different levels of CAPE depression symptoms (*N* = 261).

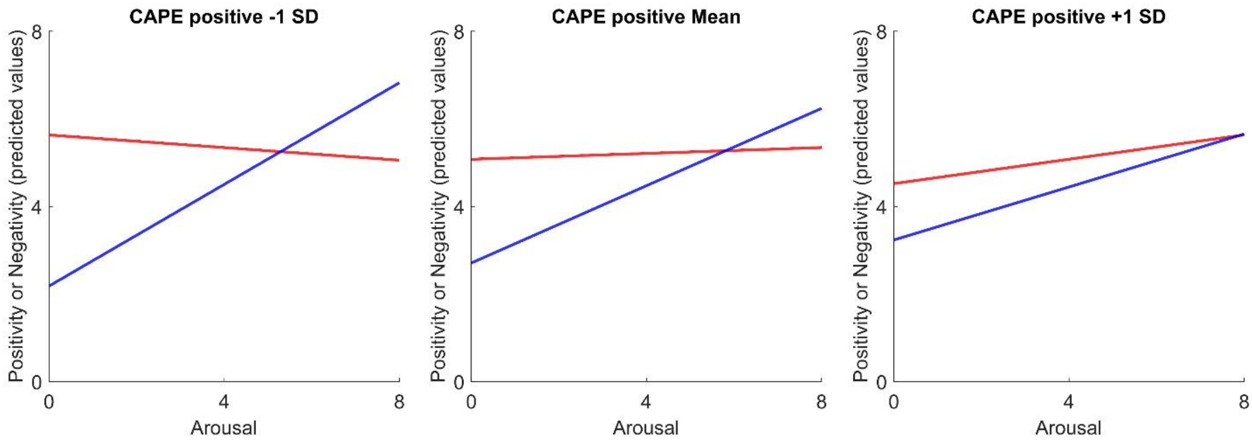

Figure S2. Positivity (red) and negativity (blue) activation functions estimated for different levels of CAPE positive symptoms (*N* = 261).

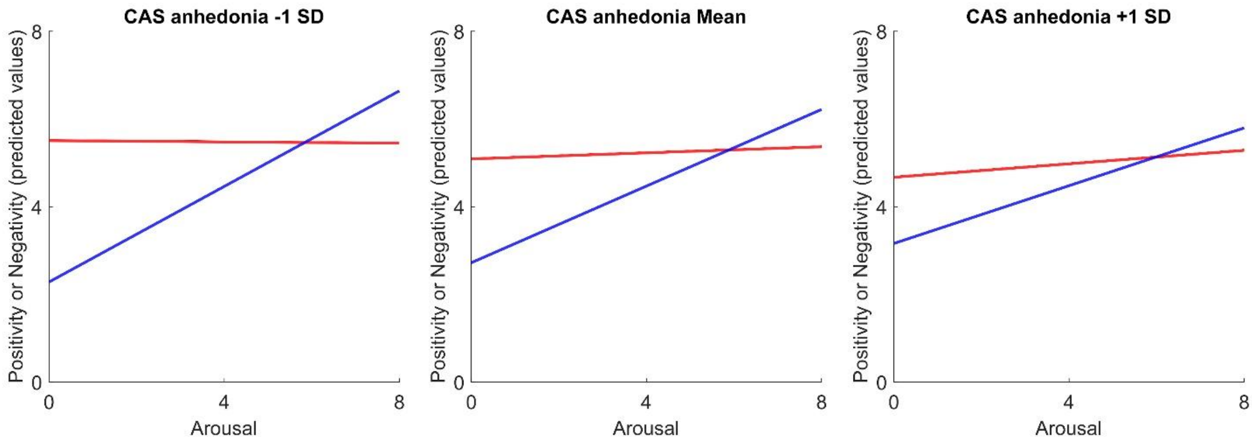

Figure S3. Positivity (red) and negativity (blue) activation functions estimated for different levels of CAS anhedonia (*N* = 261).
